# Estimating the prevalence and predictors of musculoskeletal disorders in Tanzania: a cross-sectional pilot study

**DOI:** 10.11604/pamj.2024.47.36.38258

**Published:** 2024-01-30

**Authors:** Nateiya Mmeta Yongolo, Jo Halliday, Christopher Bunn, Benson Mtesha, Clive Kelly, Stefanie Jennifer Krauth, Anthon Mwingwa, Sanjura Mandela Biswaro, Stefan Siebert, Asia Hemed Kipengele, Richard William Walker, Emma McIntosh, Blandina Theophil Mmbaga

**Affiliations:** 1 Kilimanjaro Clinical Research Institute (KCRI), Moshi, Tanzania,; 2 School of Health and Wellbeing, University of Glasgow, Glasgow, United Kingdom,; 3 Liverpool School of Tropical Medicine, Liverpool, England,; 4 School of Biodiversity, One Health and Veterinary Medicine, University of Glasgow, Glasgow, United Kingdom,; 5 Population Health Sciences Institute, Newcastle University, Newcastle upon Tyne, United Kingdom,; 6 School of Infection and Immunity, University of Glasgow, Glasgow, United Kingdom,; 7 Kilimanjaro Christian Medical University College, Moshi, Tanzania

**Keywords:** Cross-sectional studies, pilot projects, Tanzania, rheumatology, prevalence

## Abstract

**Introduction:**

musculoskeletal (MSK) disorders account for approximately 20% of all years lived with disability worldwide however studies of MSK disorders in Africa are scarce. This pilot study aimed to estimate the community-based prevalence of MSK disorders, identify predictors, and assess the associated disability in a Tanzanian population.

**Methods:**

a cross-sectional study was conducted in one village in the Kilimanjaro region from March to June 2019. The Gait, Arms, Legs, Spine (GALS) or paediatric GALS (pGALS) examinations were used during household and school visits. Individuals positive in GALS/pGALS screening were assessed by the regional examination of the musculoskeletal system (REMS) and Modified Health Assessment Questionnaire (MHAQ).

**Results:**

among the 1,172 individuals enrolled in households, 95 (8.1%, 95% CI: 6.6 - 9.8) showed signs of MSK disorders using the GALS/pGALS examination and 37 (3.2%, 95% CI: 2.2 - 4.3) using the REMS. Among 682 schools enrolled children, seven showed signs of MSK disorders using the GALS/pGALS examination (1.0%, 95% CI: 0.4 - 2.1) and three using the REMS (0.4%, 95% CI: 0.0 - 1.3). In the household-enrolled adult population, female gender and increasing age were associated with GALS and REMS-positive findings. Among GALS-positive adults, increasing age was associated with REMS-positive status and increasing MHAQ score.

**Conclusion:**

this Tanzanian study demonstrates a prevalence of MSK disorders and identifies predictors of MSK disorders comparable to those seen globally. These findings can inform the development of rheumatology services and interventions in Tanzania and the design of future investigations of the determinants of MSK disorders, and their impacts on health, livelihoods, and well-being.

## Introduction

The burden of non-communicable diseases (NCDs) is a global health concern [1]. Among NCDs, musculoskeletal (MSK) disorders are significant contributors to disability [2], accounting for 20% of all years lived with disability (YLDs) in low and middle-income countries (LMICs) [3]. The prevalence of MSK disorders is influenced by many factors including genetics, trauma, infection, autoimmunity, and lifestyle [4,5]. In most global settings MSK disorders are more prevalent in women and older age groups [6]. Religion and ethnic groups have also been associated with MSK disorders [7]. Musculoskeletal disorders occur globally but access to appropriate diagnostic and management services in LMICs is minimal [8]. There are limited rheumatological services in East Africa [9] and no rheumatologists in Northern Tanzania (personal communication with the registrar of the Medical Council of Tanganyika). The lack of diagnosis and management of MSK disorders negatively impacts sufferers, increasing the risks of many NCDs, adversely affecting psychosocial status, quality of life, household income, and well-being and potentially leading to permanent disability [1,10,11].

Screening tools to detect MSK disorders provide a standard screening approach that is practical for both patients and clinicians. The gait, arms, legs and spine (GALS) (ages ≥18 years) and paediatric GALS (pGALS) (ages ≤18 years) screening tools, and the Regional Examination of the Musculoskeletal System (REMS) are short physical examinations which have been validated for detecting MSK disorders [12-19]. The Health Assessment Questionnaire (HAQ) is used to assess physical disability associated with MSK disorders [20] and the modified HAQ (MHAQ) was developed for use in community settings [20].

Data on the prevalence of MSK disorders in LMICs are limited, with no published data to date from Tanzania. This pilot study applied the GALS/pGALS, REMS, and MHAQ tools with minor modifications to provide initial estimates of the prevalence of MSK disorders in Tanzania. These data will inform the development of much-needed rheumatology services in Tanzania as well as the design of future studies in rural Tanzania. This study is intended to answer the following research questions.

Research questions: 1) what is the prevalence of MSK disorders (defined as GALS/pGALS and REMS positive status) in Kimira village, Hai District Tanzania? 2) What is the physical disability impact (defined by MHAQ score) associated with MSK disorders in Kimira village, Hai District Tanzania? 3) What are the predictors associated with MSK disorders in Kimira village, Hai District, Tanzania?

## Methods

**Study design:** this community-based pilot study was conducted between March and June 2019 in a single village within the Hai District of Kilimanjaro region, Tanzania.

**Study setting and participants:** the village is situated on the southern slopes of Kilimanjaro at an elevation of 1,100 M. Initially, meetings with village leaders and enumerators (employed for the 2012 population census) were held to introduce the project. A list of all households was obtained from enumerators and all household members were invited to participate in the study. In addition, all schools within the village´s geographical boundaries were visited to gather data from children. Inclusion criteria for participation in the study were: age five years or greater and village residency. When individuals were not available during the visit, another time was arranged. At school visits, children were provided with an information sheet for their parents to review.

**Data sources and measurement:** to determine the prevalence of MSK disorders, GALS or pGALS examination was performed for all participants [14]. GALS/pGALS was used to detect the presence and region in the body with physical signs of MSK disorders. Questions were translated into Swahili, and extra questions were adapted for the rural Tanzanian context (Annex 1). Participants were classified as positive for GALS/pGALS when they presented with one or more objective physical signs in the gait, arms, legs, or spine.

All individuals classified as positive by GALS/pGALS screening underwent further examination using REMS [12]. Regional examination of the musculoskeletal system was performed on the regions identified in the GALS examination to get detailed clinical signs and data on the functionality of the regions affected by MSK disorders. Participants were classified as REMS positive if they exhibited one or more MSK abnormalities in the examination. To determine the physical disability impact of MSK disorders, the MHAQ was administered for all GALS/pGALS positive participants. Questions were adapted for the Tanzanian context as well as being translated into Swahili (Annex 1). All questions were asked with a three-month recall period. Respondents´ total scores were calculated to provide an overall MHAQ classification [20].

**Variables and data collection tool:** physical examinations (GALS and REMS examinations) were performed by trained Tanzanian registered medical doctors or nurses. Data on participant age, sex, tribe, and religion were gathered and recorded using Open Data Kit (ODK) software, and then uploaded to secure servers for data management, analysis, and storage. All interviews were conducted in Swahili.

**Data analysis:** all analyses were performed using R statistical software [21]. Records with incomplete data were excluded from analyses due to the likelihood of selection bias in the data gathered from school children with high levels of incomplete data. For descriptive analysis (prevalence and demographic characteristics: age, gender, religion, and tribe), all calculated proportions were reported with exact binomial confidence intervals. To determine predictors associated with MSK disorders and the physical disability impact, regression analyses were performed. Regression analyses were used to identify variables associated with the outcome variables: GALS/pGALS status, REMS status, and MHAQ scores. Independent variables evaluated for all models were participant age (in years), gender, and religion. Multivariable models were constructed for each outcome variable. The models with GALS/pGALS and REMS status outcomes were fitted using data from household-enrolled adult participants. Models with REMS status and MHAQ score outcomes were fitted using data from the GALS-positive adult household enrolled population. To confirm the statistical significance of associations assessed for each outcome an initial maximal model was built and model simplification was performed using likelihood ratio tests (LRT) with a threshold of p<0.05.

**Ethical considerations:** the study was approved by the College of Medical, Veterinary and Life Sciences (MVLS) Ethics Committee at the University of Glasgow (200180100), the Kilimanjaro Christian Medical University College Local Ethical Review Committee (KCMC/P.I/Vol.XI/2407) and the National Institute for Medical Research in Tanzania (NIMR/HQ.R.8a/Vol.IX/3038). Written informed consent for all participants was obtained, whereby all adults aged 18 years and above were provided with an information sheet and signed a consent form. For adult participants who had challenges to read and write, research assistants read the information sheet to them and they signed a consent form while being witnessed by a relative or guardian. Children aged 5 years to 17 years were asked to give assent and legal guardians signed a consent form on their behalf.

## Results

**Descriptive analysis (prevalence and demographic characteristics):** a total of 1,854 participants were enrolled, with 1,172 (63.2%) from households and 682 (36.8%) from schools. Details of the sampled populations and outcome measures are shown in Table 1 and Table 2. Details of the reasons for non-participation of some approached participants were not provided but not all respondents approached agreed to participate. Of the 1,172 household enrolled individuals, 95 (8.1%, 95% CI: 6.6 - 9.8) were GALS/pGALS positive, and 37 (3.2%, 95% CI: 2.2 - 4.3) were also REMS positive. Of the 826 household enrolled adults, 91 (11.0%, 95% CI: 9.0 - 13.4) were GALS positive, and 36 (4.4%, 95% CI: 3.1 - 6.0) were also REMS positive. Of the 346 household enrolled minors, four (1.2%, 95% CI: 0.03 - 2.9) were pGALS positive, and one (0.03 %, 95% CI: <0.01 - 1.6) was REMS positive. Of the 682 school-enrolled individuals, eight were aged 18 years or older and were assessed by GALS but none were positive. Of the remaining enrolled, 674 minors enrolled in schools, seven (1.0%, 95% CI: 0.4 - 2.1) were pGALS positive and three (0.4%, 95% CI: <0.01 - 1.3) were REMS positive.

**Table 1:** summary of household (n=1172) and school enrolled (n=682) participants with GALS/pGALS and REMS positive status by age group, gender, religion, and tribe

Variable	Levels	Household		School	
		GALS or pGALS positive/ total assessed n/N (%)	REMS positive/GALS or pGALS positive n/N (%)	GALS or pGALS positive/ total assessed n/N (%)	REMS positive/GALS or pGALS positive n/N (%)
**Gender**					
	Male	23/508 (4.5%)	6/23 (26.1%)	2/295 (0.7%)	1/2 (50.0%)
	Female	72/664 (10.8%)	31/72 (43.0%)	5/387 (1.3%)	2/5 (40.0%)
**Age (years)**					
	5-17	4/346 (1.2%)	1/4 (25.0%)	7/674 (1.0%)	3/7 (42.9%)
	18-24	1/97 (1.0%)	0/1 (0%)	0/8 (0%)	0 (0%)
	25-34	2/119 (1.7%)	0/2 (0%)	-	-
	35-44	6/140 (4.3%)	2/6 (33.3%)	-	-
	45-54	10/157 (6.4%)	1/10 (10.0%)	-	-
	55-64	21/138 (15.2%)	10/21 (47.6%)	-	-
	65 above	51/175 (29.1%)	22/51 (43.1%)	-	-
**Religion**					
	Christian	77/960 (8.0%)	28/77 (36.4%)	0/167 (0%)	0 (0%)
	Muslim	17/186 (9.1%)	9/17 (52.9%)	0/81 (0%)	0 (0%)
	NA	26		434	
**Tribe**					
	Chaga	95/1093 (8.7%)	37/95 (38.9%)	0/228 (0%)	
	Others	0/57 (0%)	0 (0%)	0/14 (0%)	
	NA	22		440	

GALS: gait, arms, legs, and spine; pGALS: paediatric GALS; REMS: regional examination of musculoskeletal system; NA: not applicable indicates number of observations with missing data

**Table 2:** number and proportion of the GALS/pGALS positive participants (n=102) with different MHAQ classifications by age groups, gender, religion, and tribe

Variable	Levels	MHAQ category n/N (%)			
		<0.3 (normal functional status)	0.3-1.29 (mild functional loss)	1.3-1.79 (moderate functional loss)	≥1.8 (severe functional loss)
**Gender**					
	Male	19/25 (76.0%)	4/25 (16.0%)	2/25 (8.0%)	0/25 (0%)
	Female	45/77 (58.4%)	24/77(31.2%)	3/77 (3.9%)	5/77 (6.5%)
**Age (years)**					
	5-17	9/11 (81.8%)	2/11 (18.2%)	0/11 (0%)	0/11 (0%)
	18-24	1/1(100%)	0/1 (0%)	0/1 (0%)	0/1 (0%)
	25-34	2/2 (100%)	0/2 (0%)	0/2 (0%)	0/2 (0%)
	35-44	5/6 (83.3%)	1/6 (16.7%)	0/6 (0%)	0/6 (0%)
	45-54	9/10 (90.0%)	1/10 (10.0%)	0/10 (0%)	0/10 (0%)
	55-64	10/21 (47.6%)	8/21 (38.1%)	2/21 (9.5%)	1/21 (4.8%)
	65 above	28/51 (54.9%)	16/51 (31.4%)	3/51 (5.9%)	4/51(7.8%)
**Religion**					
	Christian	49/77 (63.6%)	19/77 (24.7%)	5/77 (6.5%)	4/77 (5.2%)
	Muslim	9/17 (52.9%)	7/17 (41.2%)	0/17 (0%)	1/17 (5.9%)
	NA	8			
**Tribe**					
	Chaga	58/95 (61.1%)	27/95 (28.4%)	5/95 (5.3%)	5/95 (5.3%)
	NA	7			

MHAQ: modified health assessment questionnaire; NA: not applicable indicates number of observations with missing data for a given variable

Of the 102 individuals (all ages) classified as GALS/pGALS positive through both household and school enrolment, 64 (62.7%) had normal functional status using the MHAQ tool, 28 (27.5%) had a mild functional loss, 5 (4.9%) had moderate functional loss and 5 (4.9%) had a severe functional loss (Table 2).

**Predictors of musculoskeletal disorders and physical disability impact:** regression analyses were restricted to the household-enrolled adult population due to the small numbers of GALS positive children. The variable ´tribe´ was excluded from all models because of the dominance of a single tribe (Chaga). The logistic regression modelling of GALS and REMS status revealed that they were significantly associated with older age and female gender (Table 3). In the GALS positive cases, increased age was associated with positive REMS status. The linear regression model for the MHAQ score revealed that increased age was associated with a higher MHAQ score in the GALS positive population (Table 3).

**Table 3:** model summaries for multivariable regression analyses of GALS status, REMS status and MHAQ score outcomes in the adult household enrolled population

Modelled outcome (population)	Age (years)		Gender (F vs M)		Pseudo R^2^
	**OR (95% CI)**	**p-value**	**OR (95% CI)**	**p-value**	
GALS status (adult household)	1.06 (1.05-1.08)	<0.001	2.45 (1.44-4.16)	0.001	0.24
REMS status (adult household)	1.08 (1.05-1.10)	<0.001	3.40 (1.36-8.53)	0.009	0.26
REMS status (adult household GALS positive)	1.04 (1.01-1.07)	0.011	-	-	0.11
**Outcome (population)**	**β(CI)**	**p-value**	**β(CI)**	**p-value**	
MHAQ score (adult household GALS positive)	0.12 (0.06-0.19)	<0.001	-	-	0.13

GALS: gait, arms, legs, and spine; REMS: regional examination of musculoskeletal system; OR: odds ratio; β: coefficient; CI: confidence interval; MHAQ: modified health assessment questionnaire

## Discussion

This pilot study, reveals an overall prevalence of GALS/pGALS positivity of 8.1% and REMS positivity of 3.2% in this rural Tanzanian household enrolled population. These estimates, generated with tools adapted for this context, indicate a burden of MSK disorders similar to the overall prevalence estimate generated by the global burden of disease study 2010 [22]. These estimates provide a strong rationale for further investigation of the clinical, economic, and societal burden of MSK in Tanzania.

The GALS/pGALS and REMS tools cannot be used alone to diagnose arthritis. However, they are valuable to help identify cases and inform further investigations. The diagnosis of specific MSK disorder requires clinical history, physical examination and additional investigations [17,19,20,23,24].

The findings revealing that age and gender were both associated with GALS and REMS status are in line with previous studies of MSK disorders [4,6,24,25]. Investigation of modifiable factors associated with MSK disorders was beyond the scope of this study. Minor revisions were made to the MHAQ tool for application in the Tanzanian population and it is possible that these changes may have influenced the classifications reported, affecting the comparability of these data with other studies. Furthermore, the results showed an association between increased age and higher MHAQ scores, reflecting a greater loss of function in older age groups, as seen in the USA [10,24].

This was a small-scale pilot study, incorporating limited variables, from a single village only and the findings cannot be used to extrapolate results to wider populations e.g. national level or all rural areas. Similarly, because the analysis in this study included only data from adults, these results cannot be extrapolated to children. This pilot study provided valuable lessons to inform future studies that can in turn be used to inform policy and guideline updates. The GALS/pGALS, REMS and MHAQ tools can be applied with minor adaption and appropriate training in rural Tanzania. The high proportions of missing data and limited GALS/pGALS positive individuals in schools indicate future studies should enrol children in their households. The GALS/pGALS tool may classify some individuals with non-MSK disorders as positive, with important implications for the interpretation of prevalence studies. The GALS/pGALS-positive population identified in this study included some individuals with neuropathic disorders and amputations. Future studies could include additional questions to enable the collection of data on possible underlying causes of GALS/pGALS positive status [24,26,27].

## Conclusion

Findings from this study provide a first indication of the prevalence of MSK disorders and possible arthritis in rural Northern Tanzania, indicating that these disorders constitute an important health problem in this community. Further research to quantify the extent and impact of MSK disorders in multiple contexts in Tanzania and sub-Saharan Africa as a whole can provide evidence of the aetiology and impact of these disorders and their effects on the lives and well-being of affected patients, their families, and communities.

### What is known about this topic

- *Musculoskeletal (MSK) diseases are a leading cause of disability globally;*
- *Older age and female gender are often associated with MSK disorders in many contexts worldwide*.

### What this study adds

- *In this rural Tanzanian household enrolled population, the prevalence of GALS/pGALS positivity is 8.1% and the prevalence of REMS positivity is 3.2%;*
- *In this rural Tanzanian population, of those GALS-positive respondents, 4.9% had a moderate functional loss, 4.9% had a severe functional loss, and increasing age is associated with a higher MHAQ score;*
- *This population does not have access to any rheumatology services and as such this study reveals MSK disorders to be an unmet health need in Tanzania*.

## Annex

**Annex 1**: summary of the examination questions and assessment activities used for the GALS, pGALS, and MHAQ tools applied in this study, including adaptations made for the Tanzanian context and Swahili translations, for the MHAQ, all questions were asked with a three-month recall period (PDF-85 Kb)

## References

[1] Briggs AM, Cross MJ, Hoy DG, Sanchez-Riera L, Blyth FM, Woolf AD (2016). Musculoskeletal Health Conditions Represent a Global Threat to Healthy Aging: A Report for the 2015 World Health Organization World Report on Ageing and Health. Gerontologist.

[2] Hoy DG, Smith E, Cross M, Sanchez-Riera L, Blyth FM, Buchbinder R (2015). Reflecting on the global burden of musculoskeletal conditions: lessons learnt from the global burden of disease 2010 study and the next steps forward. Ann Rheum Dis.

[3] Murray CJ, Vos T, Lozano R, Naghavi M, Flaxman AD, Michaud C (2012). Disability-adjusted life years (DALYs) for 291 diseases and injuries in 21 regions, 1990-2010: a systematic analysis for the Global Burden of Disease Study 2010. Lancet.

[4] Usenbo A, Kramer V, Young T, Musekiwa A (2015). Prevalence of Arthritis in Africa: A Systematic Review and Meta-Analysis. PLoS One.

[5] Arend WP (1997). The pathophysiology and treatment of rheumatoid arthritis. Arthritis Rheum.

[6] Brennan-Olsen SL, Cook S, Leech MT, Bowe SJ, Kowal P, Naidoo N (2017). Prevalence of arthritis according to age, sex and socioeconomic status in six low and middle income countries: analysis of data from the World Health Organization study on global AGEing and adult health (SAGE) Wave 1. BMC Musculoskelet Disord.

[7] Chokkhanchitchai S, Tangarunsanti T, Jaovisidha S, Nantiruj K, Janwityanujit S (2010). The effect of religious practice on the prevalence of knee osteoarthritis. Clin Rheumatol.

[8] Joshipura M, Gosselin RA (2020). Surgical Burden of Musculoskeletal Conditions in Low-and Middle-Income Countries. World J Surg.

[9] Genga EK, Moots RJ, Oyoo OG, Otieno FO (2017). Building a rheumatology team for East Africa: A call for action!. Rheumatology (Oxford).

[10] Barbour KE, Helmick CG, Boring M, Brady TJ (2017). Vital Signs: Prevalence of Doctor-Diagnosed Arthritis and Arthritis-Attributable Activity Limitation-United States 2013-2015. MMWR Morb Mortal Wkly Rep.

[11] Olivier N, Burger J, Joubert R, Lubbe M, Naude A, Cockeran M (2018). Chronic disease list conditions in patients with rheumatoid arthritis in the private healthcare sector of South Africa. Rheumatol Int.

[12] Coady D, Walker D, Kay L (2004). Regional Examination of the Musculoskeletal System (REMS): a core set of clinical skills for medical students. Rheumatology (Oxford).

[13] Plant MJ, Linton S, Dodd E, Jones PW, Dawes PT (1993). The GALS locomotor screen and disability. Ann Rheum Dis.

[14] Doherty M, Dacre J, Dieppe P, Snaith M (1992). The 'GALS' locomotor screen. Ann Rheum Dis.

[15] Lillicrap MS, Byrne E, Speed CA (2003). Musculoskeletal assessment of general medical in-patients--joints still crying out for attention. Rheumatology (Oxford).

[16] Hood C, Johnson J, Kelly C (2001). What is the prevalence of rheumatic disorders in general medical inpatients?. Postgrad Med J.

[17] Beattie KA, Bobba R, Bayoumi I, Chan D, Schabort I, Boulos P (2008). Validation of the GALS musculoskeletal screening exam for use in primary care: a pilot study. BMC Musculoskelet Disord.

[18] Foster HE, Jandial S (2013). pGALS-paediatric Gait Arms Legs and Spine: a simple examination of the musculoskeletal system. Pediatr Rheumatol Online J.

[19] Smith E, Molyneux E, Heikens GT, Foster H (2012). Acceptability and practicality of pGALS in screening for rheumatic disease in Malawian children. Clin Rheumatol.

[20] Maska L, Anderson J, Michaud K (2011). Measures of functional status and quality of life in rheumatoid arthritis: Health Assessment Questionnaire Disability Index (HAQ), Modified Health Assessment Questionnaire (MHAQ), Multidimensional Health Assessment Questionnaire (MDHAQ), Health Assessment Questionnaire II (HAQ-II), Improved Health Assessment Questionnaire (Improved HAQ), and Rheumatoid Arthritis Quality of Life (RAQoL). Arthritis Care Res (Hoboken).

[21] R Core Team The R Project for Statistical Computing.

[22] Smith E, Hoy DG, Cross M, Vos T, Naghavi M, Buchbinder R (2014). The global burden of other musculoskeletal disorders: estimates from the Global Burden of Disease 2010 study. Ann Rheum Dis.

[23] Australian Rheumatology Association - Quality and Safety Sub-committee (2010). Modified Health Assessment Questionnaire (MHAQ).

[24] Uhlig T, Slatkowsky-Christensen B, Moe RH, Kvien TK (2010). The burden of osteoarthritis: the societal and the patient perspective. Therapy.

[25] Wijnhoven HA, de Vet HC, Picavet HS (2006). Prevalence of musculoskeletal disorders is systematically higher in women than in men. Clin J Pain.

[26] Badley EM, Canizares M, Perruccio AV (2017). Population-Based Study of Changes in Arthritis Prevalence and Arthritis Risk Factors Over Time: Generational Differences and the Role of Obesity. Arthritis Care Res (Hoboken).

[27] Brennan-Olsen SL, Solovieva S, Viikari-Juntura E, Ackerman IN, Bowe SJ, Kowal P (2018). Arthritis diagnosis and symptoms are positively associated with specific physical job exposures in lower-and middle-income countries: cross-sectional results from the World Health Organization's Study on global AGEing and adult health (SAGE). BMC Public Health.

